# On the Functional and Extra-Functional Properties of IMU Fusion Algorithms for Body-Worn Smart Sensors

**DOI:** 10.3390/s21082747

**Published:** 2021-04-13

**Authors:** Nils Büscher, Daniel Gis, Volker Kühn, Christian Haubelt

**Affiliations:** 1 Institute of Applied Microelectronics and Computer Engineering, Faculty of Computer Science and Electrical Engineering, University of Rostock, 18051 Rostock, Germany; daniel.gis@uni-rostock.de (D.G.); christian.haubelt@uni-rostock.de (C.H.); 2 Institute of Communications Engineering, Faculty of Computer Science and Electrical Engineering, University of Rostock, 18051 Rostock, Germany; volker.kuehn@uni-rostock.de

**Keywords:** sensor fusion, fixed-point arithmetic, hardware restrictions, AHRS algorithms, human interaction

## Abstract

In this work, four sensor fusion algorithms for inertial measurement unit data to determine the orientation of a device are assessed regarding their usability in a hardware restricted environment such as body-worn sensor nodes. The assessment is done for both the functional and the extra-functional properties in the context of human operated devices. The four algorithms are implemented in three data formats: 32-bit floating-point, 32-bit fixed-point and 16-bit fixed-point and compared regarding code size, computational effort, and fusion quality. Code size and computational effort are evaluated on an ARM Cortex M0+. For the assessment of the functional properties, the sensor fusion output is compared to a camera generated reference and analyzed in an extensive statistical analysis to determine how data format, algorithm, and human interaction influence the quality of the sensor fusion. Our experiments show that using fixed-point arithmetic can significantly decrease the computational complexity while still maintaining a high fusion quality and all four algorithms are applicable for applications with human interaction.

## 1. Introduction

Inertial sensors using the Micro-Electro-Mechanical Systems (MEMS) technology have become the de-facto standard for inertial measurement units (IMU) in consumer electronics [1]. Furthermore, due to their capabilities and energy efficiency, they are also predestined for gesture- and activity recognition, health monitoring [2], smart clothes, or remote devices powered through energy harvesting.

For the named applications, oftentimes, smart sensor hubs or sensor nodes (In the following, we use the term smart sensor to refer to these types of devices) are used which incorporate not only the inertial sensors but also a microcontroller (μC) and interfaces to preprocess, fuse, and analyze the captured data [3]. Integrating all functions in a System in Package (SiP) yields many benefits. The preprocessing and sensor fusion can directly be done on the smart sensor, which can result in a reduced communication overhead and the possibility to have independently working components that can easily be used for different purposes.

Additionally, a further decrease in power consumption is possible depending on the specific application. Taking, for example, a body worn sensor with a 100 mAh battery that measures and transmits data via Bluetooth Low Energy (BLE) and consumes 12 mA. If a preprocessing can reduce the required supply current of the transmission by 3 mA and increase consumption of the μC by only 1 mA, the overall system would consume 2 mA less and therefore the sensor can run 100 min longer before the battery is depleted.

However, regardless of the system architecture, the used μCs are often limited regarding their computational power and memory size to reduce the power consumption. For example, a Cortex M0+ requires up to 5.9 mA [4] while a more powerful Cortex M3 requires up to 27 mA [5]. In particular, complex sensor fusion algorithms with a high Output Data Rate (ODR) can easily reach or exceed the limits of the smaller μC.

In this work, we study the suitability of four widely used sensor fusion algorithms to determine the orientation of a body-worn device in a hardware restricted environment and the aim for a low power consumption.

Our experiments show that using fixed-point arithmetic for the sensor fusion can drastically reduce the computational effort while still rendering the algorithms usable in most cases. Furthermore, we observed that the memory requirements are not affected significantly by the different data formats. To determine how much the used algorithms and data formats influence the quality of the fusion results, an extensive statistical Gage R&R analysis has been conducted. Moreover, as reference, we used a camera-based approach to determine the true performed motion. The results of the analysis showed that all filters are applicable for the usage in devices with human interaction.

This work is organized as follows:

After discussing related work in Section 2, background information about the used hardware and algorithms as well as details about the data formats and statistical analysis method are given in Section 3. Afterwards, in Section 4, the extra-functional properties’ code size and computational effort are evaluated. Subsequently, Section 5 covers first a general analysis of the sensor fusion results and then a statistical analysis of the sensor fusion results. Finally, Section 6 concludes the findings of this work.

## 2. Related Work

The work at hand uses four sensor fusion algorithms to determine the orientation of a device. These types of methods are often referred to as Attitude and Heading Reference System (AHRS) algorithms commonly used in conjunction with aviation or aerial vehicles [6,7]. The evaluation has been conducted with an extended Kalman filter, the Madgwick filter [8,9], the Mahony filter [10], and a complementary filter [11].

In the past, there have already been multiple studies which compared the computed fusion results of these algorithms quantitatively. For instance, in [12], a comparison between Kalman filter, Mahony filter, and Madwick filter is coming to a similar conclusion as us that the Madwick filter and Manhony filter are well usable in hardware limited environments. Evaluations are both done in a simulation and with real recorded data.

Other works confirming the usability of the complementary filter based sensor fusion in comparison to a Kalman filter are shown in [13,14]. Both works compare a Kalman filter and a complementary filter and conclude that both types of filters are usable and comparable. In [13], it is stated that the complementary filter is easier to calibrate because it has fewer parameters. Both works carry out a direct comparison of two or more sensor fusion algorithms for the orientation and come to the consistent conclusion that the complementary filter based approaches are not inferior to the Kalman filters.

In contrast to the previous mentioned works, it is concluded in [15] that the Kalman filter is superior compared to the Madgwick or the Mahony filter. The comparison in [15] is done for multiple movements speeds using a robotic arm as a reference. Although it is concluded that the Kalman filter is better, the differences of the measurements are quite small. The error of Madgwick filter and Mahony filter are on average only 0.4° higher than the error from the Kalman filter for an overall average error of 3.4°. The differences are quite likely due to the usage of a different measurement system including different assumptions about the conducted movement speeds and scenarios. Additionally, the impact of using a robotic arm with very abrupt but linear movements does not allow a direct conclusion about the filter behavior when the movement is conducted by humans. In the work at hand, the movements are more fluent and more diverse than with a robot.

In comparison to our study, the above-mentioned papers do not investigate different data formats. Moreover, they do not conduct a statistical analysis on the filter quality, which is of particular importance when studying natural movements. Finally, they do not directly analyze extra-functional properties like computational effort and codes size as it is done in our work at hand.

Other works examine the usability of complementary filter based approaches to be used in body-worn sensory and health care applications. This emphasizes the need for algorithms with low hardware requirements and the usability of complementary filter based approaches. In [16], a novel approach for a complementary filter is presented especially tailored for the usage in body-worn sensory with limited hardware capabilities. An evaluation has been done with a motion capturing system to determine the precision of the proposed algorithm. In conclusion, Ref. [16] states that the proposed complementary filter is usable for the usage in body-worn sensors, which confirms the findings of the work at hand. Furthermore, in [17], a head-mounted system for fall detection has been proposed using the Madgwick filter to calculate the orientation of the head mounted device. The authors state that the Madwick filter is used because of its lower computational effort and the fact that it does work well with low update frequencies and does not need an initialization phase. However, in contrast to our work, these two approaches again do not systematically study extra-functional properties.

## 3. Background and Methods

Before the evaluation of the functional and extra-functional properties of the sensor fusion algorithms are described in Section 4 and Section 5, this section will provide general information about the used sensor fusion algorithms, data formats, hardware, and the implementation. This information is viable to put the results and interpretations in the correct context. Additionally, these sections describe the methods used to assess the extrafunctional properties and functional properties of the fusion algorithms.

### 3.1. Used Algorithms

For the investigation of the AHRS sensor fusion algorithms, the four most widely used algorithms to determine the orientation of a device, namely the Madgwick filter, the Mahony filter, an extended Kalman filter and the complementary filter, have been chosen. The implementations of the extended Kalman filter as well as the complementary filter use the method described in [18] to determine the orientation from accelerometer data and magnetometer data to avoid the usage of computationally expensive trigonometric functions.

Complementary Filter

The complementary filter is the most basic filter used in this work. It takes advantage of the fact that the data from the gyroscope are more precise in higher frequencies and the data from the accelerometer are more precise in lower frequencies. The complementary filter applies a low-pass filter to the orientation calculated from the accelerometer data and a high-pass filter to the orientation calculated from the gyroscope data [11]. Both orientations are then combined via a weighted addition seen in Equation (1):

(1) $$q_{t}=\left(1-\alpha \right)\cdot q_{acc}-\alpha \cdot q_{gyr}$$

The orientation quaternion qacc is extracted from the data of the accelerometer using the method described in [18]. The orientation $q_{gyr}$ is the last orientation $q_{t-1}$ updated by the current angle rates of the gyroscope. This filter uses single parameter α that is used to define the weighting between accelerometer data and gyroscope data.

Mahony Filter

The Mahony Filter is an enhancement of the complementary filter by applying a proportional-integral controller (PI controller) to the error function between the orientation from the accelerometer data and the orientation from the gyroscope data. Therefore, the Mahony filter provides good results while still being computationally inexpensive. The integral part of the controller is able to reliably remove a constant offset caused by biased accelerometer or gyroscope data. This filter has two parameters to tune the results, the factor for the proportional part $K_{p}$ and factor for the integral part $K_{i}$. The proportional and integrated errors are calculated for each axis of the gyroscope input. The correction from the PI Controller is also applied to the gyroscope input data. The correction step can be seen in Equation (2), which shows the correction of the angle rate for one of the three axes:

(2) $$anglerate_{corrected}=anglerate+error_{p}\cdot K_{p}+error_{i}$$

(3) $$error_{i}=error_{i}+error_{p}\cdot K_{i}\cdot dt$$

The proportional error $error_{p}$ is calculated as the sum of the cross product between the gravity vector estimated by the orientation that was updated by the gyroscope data and the gravity vector measured by the accelerometer. The integral error $error_{i}$ is calculated using Equation (3) by integrating the proportional error $error_{p}$ multiplied with $K_{i}$ and the sampling period dt.

The corrected angle rate $anglerate_{corrected}$ is then used to update the current orientation.

Madgwick filter

The Madgwick filter is also loosely based on the concepts of the complementary filter but uses a gradient descent algorithm to calculate the orientation error and fuse the orientation from the accelerometer data and the orientation from the gyroscope data. This filter uses a single β parameter used for the steepness of the gradient descent algorithm. The correction of the orientation using the β parameter is shown in Equation (4):

(4) $$q_{t}=q_{t-1}+\left(update_{gyr}-\beta \cdot S\right)\cdot dt$$

The value $update_{gyr}$ is the rate of change quaternion calculated from the gyroscope data. The corrective step quaternion *S* is calculated from the current orientation and the data from the accelerometer using a gradient descent algorithm. The corrected update step for the orientation is multiplied by the sampling period dt before being added to the last orientation $q_{t-1}$.

Kalman Filter

The Kalman filter is the most computationally expensive filter used in this investigation. Theoretically, the Kalman filter is an optimal error estimator for linear problems with Gaussian noise [19,20]. For its calculation, the Kalman filter uses multiple matrices and vectors. The vector x contains the state of the system, while the matrix *P* contains the co-variance matrix for the state variables. The matrices *A* and *H* describe the dynamics of the system. The matrix *A* is the state transition matrix to predict the next step. In the case of an extended Kalman filter, this matrix has to be calculated at every update step using a linearization method. The matrix *H* describes the sensor input. The matrix *R* describes the measurement noise and the matrix *Q* describes the noise of the system. Both *R* and *Q* are the main matrices used to calibrate the Kalman filter.

The Kalman filter is a recursive filter which works in two steps. In the first step, the current state is predicted using the previous state variables and their uncertainties. The prediction step is shown in Equations (5) and (6):

(5) $$x_{t}^{-}=x_{t-1}\cdot A$$

(6) $$P_{t}^{-}=A\cdot P_{t-1}\cdot A^{T}+Q$$

The state vector $x_{t}^{-}$ contains the prediction of the next state. The matrix $P_{t}^{-}$ is the predicted co-variance of the next state.

The prediction phase is followed by the correction phase described in Equations (7)–(9):

(7) $$K_{t}=P_{t}^{-}\cdot H\cdot {\left(H\cdot P_{t}^{-}\cdot H^{T}+R\right)}^{-1}$$

(8) $$x_{t}=x_{t}^{-}+K\cdot \left(z_{t}-H\cdot x_{t}^{-}\right)$$

(9) $$P_{t}=\left(I-K_{t}\cdot H\right)\cdot P_{t}^{-}$$

In $K_{t}$ is the Kalman gain of the current step. The Kalman gain $K_{t}$ calculated in Equation (7) is used in Equation (8) to calculate the next state using the predicted state $x_{t}^{-}$ and the sensor input vector $z_{t}$. The co-variance $P_{t}$ is calculated in Equation (9) using the predicted co-variance matrix $P_{t}^{-}$ and the Kalman gain $K_{t}$. The matrix *I* is a unit matrix.

### 3.2. Quaternion Representation

All algorithms described in Section 3.1 estimate the orientation of the inertial sensor system using the quaternion representation. Quaternions are widely used in sensor fusion, computer graphics, and navigation. Other commonly used representations are Euler angles, rotation matrices, or axis-angle.

Compared to rotation matrices, the quaternion representation needs fewer values to represent a rotation. When used for sensor fusion, a key benefit of quaternions is the existence of methods to smoothly interpolate between two orientations via linear interpolation and the more precise spherical linear interpolation (SLERP) [21].

Euler angles suffer from gimbal lock or ambiguities [22]. This problem is not apparent for quaternions. Additionally, in contrast to Euler angles, the quaternions also do not require trigonometric functions for their computation, making them suitable for the usage on small μC.

Another important benefit of quaternions is the fact that they are normalized to a length of 1 to represent an orientation. Knowing the range of values of the input data, it is possible to determine the required position of the radix point to avoid overflows and retain the highest possible precision when using fixed-point arithmetic. Mathematically, quaternions can be seen as a four element vector q=(w,x,y,z) with the scalar part *w* and the values *x*, *y* and *z* representing the rotation. For later use in this work, $q_{w}$ will be defined as the *w* element of the quaternion *q*.

### 3.3. Data Formats

Depending on the target hardware the used data format can have a significant influence on the computational complexity of an algorithm. This is especially the case for floating-point data used on hardware that does not natively support floating-point operations. This is particularly true for the hardware used in this work as described below in Section 3.4. In the following, we describe the data formats used in our study.

#### 3.3.1. Single Precision Floating-Point

By default, most of the sensor fusion algorithms are implemented using a floating-point data representation. This is also the case for the four examined filters. Floating-point data have the benefit of being able to both store very large and very small numbers using a combination of an exponent and a fraction. Therefore, floating-point numbers have significantly higher ranges than fixed-point numbers of the same bit-length. Single precision floating-point numbers have an 8-bit exponent and a fraction of 23 bits, which is the limit for their precision. The downside of floating-point numbers is that they need more effort for all arithmetic operations. Even for a simple addition, both values have to be first transformed to have the same exponent before the addition can be done [23]. Afterwards, the result of the addition has to be normalized, otherwise the precision of the data would degenerate with every arithmetic operation. This increased complexity makes floating-point numbers difficult to use in a hardware restricted environment.

#### 3.3.2. Fixed-Point Numbers

As the name suggests, fixed-point data have the radix point fixed at a certain bit position, independent of the value that is stored in the fixed-point number. Their biggest advantage compared to the floating-point numbers is the lower computational effort needed for mathematical operations. In particular, additions and subtractions are very efficient. Multiplications need additional shift and optional rounding operations but are still more efficient than floating-point multiplications [24]. The downside of fixed-point arithmetic is that the range of data and precision of the data are reciprocal to each other. A higher range of the stored numbers results in a lower precision and vice versa. Using fixed-point numbers is the preferred way for a hardware restricted environment, such as smart sensors. Due to the interplay of range and precision, it has to be evaluated if fixed-point numbers are applicable for the targeted scenario.

### 3.4. Used Hardware

The hardware used for the measurement is a BMF055 smart sensor by Bosch Sensortec [25]. The BMF055 uses the SAM D20 microcontroller, an ARM Cortex M0+ μC from Atmel running at a frequency of 48 MHz [4]. It has a 32-bit architecture with two pipeline stages and is optimized for low power consumption. Important for the evaluation of the extra-functional properties of the algorithms are two key properties of the sensor:

- The SAM D20 contains a single cycle hardware multiplier for 32-bit integer numbers, which means that addition and multiplication take the same time.
- The SAM D20 does not have hardware support for floating-point numbers. All floating-point operations have to be emulated in software resulting in higher execution time and power consumption.

The gyroscope of the BMF055 is similar to the BMI055 with a range of up to ±2000°/s and a data width of 16-bit [25,26]. The accelerometer is similar to the BMA280 with a maximal range of ±16 g and a data width of 14-bits [25,27]. The magnetometer is similar to the BMM150 with resolution of 0.3 μT and a data width of 13-bits [25,28].

The μC requires a supply current of around 1 mA while idling and up to 5.9 mA under full load. The whole BMF055 requires a supply current of up to 13.7 mA when using all sensors with an ODR of 100 Hz in normal mode and an average of 2.6 mA when running in low power mode.

### 3.5. Analysis of Extra-Functional Properties

The analysis of the extra-functional properties has been conducted on the real hardware described in Section 3.4. The extra-functional properties are separated into the code size, including RAM and ROM memory and the computational effort, measured as the computation time needed to update the orientation with new sensor data.

#### 3.5.1. Code Size

For the evaluation of the extra-functional properties, the code size of the algorithms and the average computation time required to update the orientation from new IMU data were analyzed for the Cortex M0+ μC described in Section 3.4. The compilation has been done with the GNU C compiler with the -O0 optimization option and release mode with -O3 set for a fair comparison.

To see how much space the four fusion algorithms need on the μC, the compilation outputs have been analyzed with the *size tool* from the GNU ARM Embedded Toolchain for the size of the binary code that is installed on the flash memory (ROM) and required size of data needed to be held in the SRAM for variables and functions stack.

#### 3.5.2. Computational Effort

**Figure 1:**
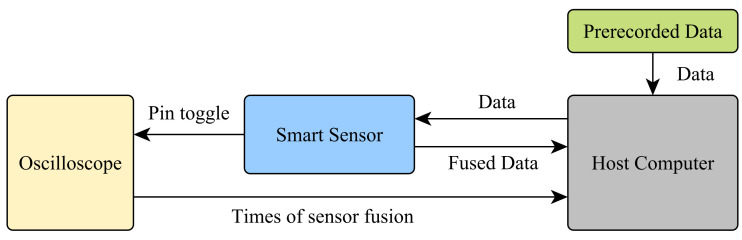
Measurement setup for the assessment of the computation time of the sensor fusion algorithms.

For the computational effort, all algorithms were executed and evaluated on a real device using the Sensor-in-the-Loop (SiL) architecture from [29] using a set of prerecorded human motion data to ensure comparable results. The software of the smart sensor has been adapted to process previously recorded data injected from a host computer via a debug interface into the μC and process it as if the data were delivered by the inertial sensors. This method makes it possible to use exactly the same data for all algorithms and data formats and thus ensures a high comparability of the measurements while still executing the software directly on the targeted hardware. The setup for the measurement can be seen in Figure 1.

To measure the computational effort of the algorithms, the host computer sends the sensor samples to the sensor. The smart sensor sets an output pin of the μC to -high- when the data arrived and the execution of the algorithm starts and resets the pin to -low- as soon as the algorithm finishes. Said pin has been connected to a high frequency oscilloscope which records the state of the pin and sends the data to the host computer for later analysis. The time required by μC to toggle a pin has been measured previously and has been taken into account for the analysis. This method allows us to measure the time the fusion algorithm requires to update the orientation for each new sensor sample. For the analysis, both the average time to update the orientation and the variance of the required time have been examined and will be discussed below.

### 3.6. Analysis of Functional Properties

**Figure 2:**
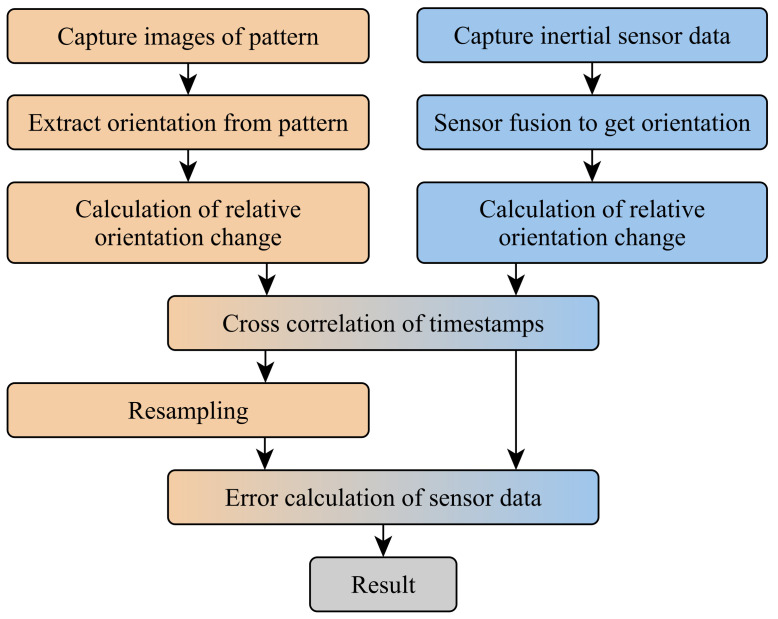
Data flow of the measurement setup. Steps involving the camera data are colored orange. Steps involving the sensor fusion result are colored blue. The resulting error is colored in gray.

The evaluation of the quality of the output of the four orientation filters was done using an external reference as ground truth against which the result of the sensor fusion was compared. The external reference was gathered using the method described in [30,31]. Said method records a pattern via a camera and uses the captured images to calculate the relative orientation between camera and pattern via image analysis using the OpenCV library [32]. A smartphone with a user interface was used to instruct the user about the desired movement and record the pattern similar to [31]. For the investigation, the smart sensor was rigidly attached to the pattern and was then moved in front of the static camera, which recorded the pattern during the measurement. It is important to note that the sensor movements are conducted by a person following an on-screen guide. That way, each experiment has been performed with similar but not identical movements. This is the reason why a sophisticated statistical analysis is done in Section 5.2. On the other hand, this way, our experiments are valid for movements that could be tracked by body-worn sensors. Figure 2 displays the steps executed to obtain and compare sensor data and reference data.

As a first step, the data from both sources were captured simultaneously and stored for the subsequent investigation. For both data sets, the relative orientation change to the start of the measurement is calculated. Using the relative orientation rather than the absolute orientation enabled us to conduct a direct comparison between the orientation without the need to transform both orientations into a common world coordinate system.

The next step of the evaluation was a cross correlation of the timestamps of the data from both sources. This step is necessary because the sensor and camera system run independently of each other and might use different time bases for their timestamps and might show a clock drift.

Finally, the reference data from the camera had to be resampled to allow for a direct comparison with the data from the sensor fusion.

The error between output orientation of the filters and the reference orientation is calculated as the minimal angle. This is the rotation around an arbitrary axis to transform the output orientation into the reference orientation. The formula to calculate the error angle can be seen in Equation (11). The result qDiff is the difference quaternion between the reference and the filter output. As shown in Equation (10), qDiff is calculated as the result of the quaternion multiplication between qRef and the conjugate of the qFilter quaternion:

(10) qDiff=qRef·conjugate(qFilter)

(11) $$error=2\cdot atan\left(qDiff_{w}\right)\cdot \frac{180}{\pi }$$

To calculate the average error between reference and sensor fusion result, the mean square error was utilized.

#### Filter and Measurement Parameters

The measurement of the sensor data has been done with a sampling frequency of 200 Hz. The image data for the reference were captured at 30 Hz. The gyroscope was configured to a sensitivity of ±2000°/s maximal angle rate resulting in a resolution of 0.061°. The accelerometer was configured to a maximal acceleration of ±16 g resulting in a resolution of 0.0048 m/s^2^.

All filters have been configured to work optimally with previously conducted calibration measurements. The parameters chosen below are described in Section 3.1. The complementary filter used an α value of 0.97. This parameter is close to an α of 0.98 often used for a complementary filter. The Mahony filter uses a value of 1.05 for the proportional part $K_{p}$ and a value of 0.2 for the integral part $K_{i}$ of the PI controller. The Madgwick filter uses a β value of 0.115. For both the Mahony filter and the Madgwick filter, the chosen values are close to the original values used from Madgwick [9]. For the Kalman filter, the process noise matrix *Q* uses a value if 0.1 for the entries in the principal diagonal. The measurement noise matrix *R* uses a value of 50.0 for the entries in the principal diagonal. The 0.1 for the R matrix was determined using the typical noise stated in the data sheet of the gyroscope [26]. The Q values were determined empirically since they depend on the expected strength of the movement done by the user.

### 3.7. Statistical Analysis

For a more in depth analysis of the influence of the different sensor fusion algorithms and data formats, a statistical analysis of the results has been conducted. For this purpose, the statistical methods used in a Gage R&R analysis have been utilized to estimate how much differences between measurements are influenced by different factors. In a traditional Gage R&R analysis, these factors are the tested part, the person testing the part, and the repetition of the measurement [33,34]. In this work, these methods have been adapted in the statistical analysis to assess the influence of the used algorithm, data format, and movement speed, respectively.

## 4. Results: Extra-Functional Properties

A key aspect of the sensor fusion algorithms that are examined in the work at hand are their extra-functional properties. These properties include the computational effort, code size, and configurability of the used sensor fusion algorithms. Especially in the context of resource limited hardware like smart sensors, the computational effort as well as the code size determine whether an algorithm can be used as is, is only usable with a limited functionality, or is not usable at all. This section will discuss the code size and computational complexity in terms of response time and throughput of the four sensor fusion algorithms implemented with the three used data formats. Before conducting the analysis of the extra-functional properties, it has been ensured that all algorithms are functional with all three data formats. An in depth analysis of the functional properties, namely the quality of the sensor fusion, will be discussed later in Section 5.

### 4.1. Code Size

**Figure 3:**
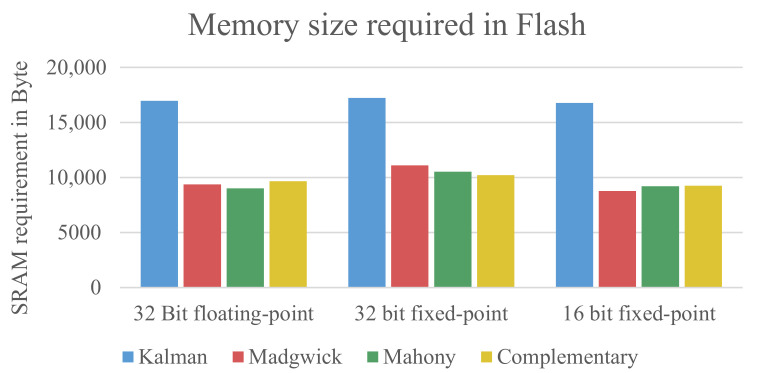
Size of data that needs to be stored in the flash memory (ROM).

The code size has been determined for each combination of fusion algorithm and data format. The results are shown and discussed in this section. Since none of the used fusion algorithms uses active memory allocation or recursive functions, the output of the compiler can directly show the needed memory size of the algorithms. The sizes of the binary file in the flash memory are displayed in Figure 3.

It can be seen that there is no huge difference between the data formats. The floating-point implementation and 16-bit fixed-point implementation are about the same size, with the 32-bit fixed-point implementation being slightly bigger. However, there is a significant difference in size between the Kalman filter and the other filters. This is mainly due to the matrix operations needed for the Kalman filter, which needs both more code and slightly more SRAM to store the values or intermediate values during the computation. The Madgwick, Mahony, and complementary filter do not require matrix operations for their computation and are therefore smaller. The matrix operations for the Kalman filter are taken from the Eigen Matrix libary [35]. The particularly complex calculation of the inverse matrix uses a specialized method for 4x4 matrices taken from the *MESA* implementation of the OpenGL Utility Libraries [36,37].

**Figure 4:**
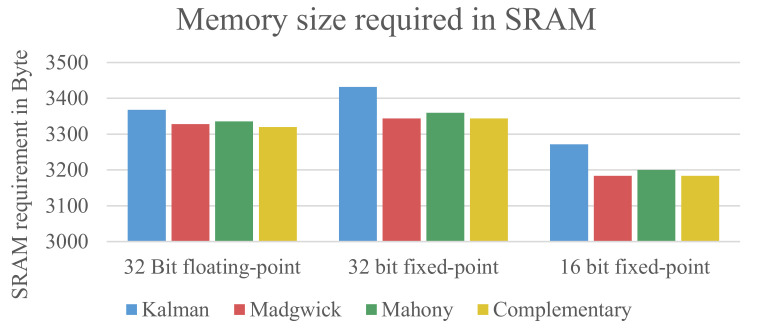
Size of data that needs to be stored in the dynamic memory (SRAM).

Figure 4 shows the size the algorithms require in the dynamic memory (SRAM) of the μC to store values, intermediate results, and the function stack.

Interestingly, the Kalman filter does not need much more dynamic storage to store the data of the matrices and intermediate results. This can be explained by the fact that Madgwick filter, Mahony Filter, and complementary filter are optimized to store many of the intermediate results in temporary variables to avoid repeated calculations for the same value when used multiple times. It can be seen that the usage of the 16-bit fixed-point data type reduces the amount of needed SRAM compared to the 32-bit floating-point implementation. The 32-bit fixed-point data type needs slightly more SRAM.

#### Explanation of the Size Differences

As described in Section 3.4, one would expect the floating-point implementation of the algorithms to need more memory than the fixed-point implementations because a SoftFloat library is needed due to the lack of hardware support. However, this is not the case because the fixed-point implementations also need additional code to operate. On the one hand, the multiplications need additional shift operations for the resulting value and a rounding of the result to reduce errors. On the other hand, an implementation of the $\frac{1}{\sqrt{x}}$ function is needed to normalize the input and output values. Said functions can be implemented very efficiently in a floating-point implementation [38,39]. The fixed-point variant needs a lookup table and therefore requires more memory. In addition to the points mentioned above, another peculiarity of fixed-point arithmetic that influences the size of the code as well as the execution time: The multiplications have to be done with twice the bit depth of the used fixed-point numbers to avoid an overflow of the intermediate result. For 16-bit fixed-point numbers, the multiplications have to be done in 32-bit, and respectively in 64-bit for 32-bit fixed-point numbers. The needed 64-bit multiplications needed for the 32-bit fixed-point implementations also explain the bigger size of the 32-bit implementation for both flash memory and SRAM. This is the case because the 64-bit operations require at least twice as many operations for the mathematical calculations and twice as many bytes to store intermediate 64-bit results.

### 4.2. Execution Time

The most important extra-functional property of the assessed fusion algorithms is the execution time, respectively the computational effort each algorithm requires to process the data from the inertial sensors. The computational effort affects both the maximal frequency with which the data can be processed and the power consumption of the μC.

**Figure 5:**
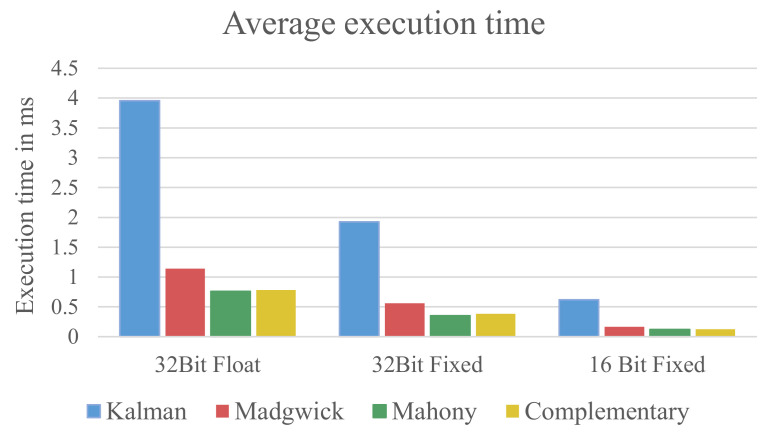
Execution time for the four algorithms grouped by data format.

The results of the measurements for the execution time of the algorithms are presented in Figure 5.

It can be clearly seen that the Kalman filter requires the highest amount of execution time for all three data formats. Compared to the other filters, the Kalman filter needs more than 3.4 times longer to compute the fusion result. For the 32-bit floating-point implementation, it needs 3.96 ms for each fusion step, which effectively limits the maximal update frequency to around 250 Hz. Considering that the μC will also have to do other calculations and control tasks, this frequency is likely to be significantly lower in the end.

The second finding is that the fixed-point implementations require only a fraction of the computation time needed by the 32-bit floating-point implementation. The 32-bit fixed-point implementation requires around 50% less time for all four algorithms than the 32-bit floating-point counterparts. Furthermore, the 16-bit fixed-point implementations require only a third of computation time required by the 32-bit fixed-point implementations.

The exact values of the execution times are listed in Table 1.

**Figure 6:**
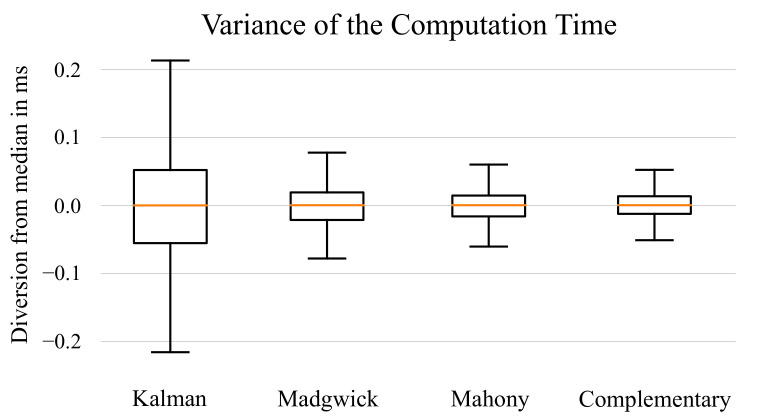
Box-Plot of the execution times for the 32-bit floating-point implementations. The boxes show the deviations from the median execution time in milliseconds.

In addition to the average execution time, the variance of the execution times have been evaluated to assess how consistent the algorithms behave. It was expected that the execution times show a low variance because the used algorithms do not contain many branches. Hence, the number of operations per sensor data update should remain nearly constant. For the fixed-point implementations, this behavior has been confirmed. However, for the floating-point implementation, the variance of the execution times was higher. In particular, the variance of the Kalman filter was significantly higher, as displayed in Figure 6.

The higher variance of the execution times for the floating-point variants has the same reason as the increased execution time in general: The mathematical operations are done inside a SoftFloat library. For additions and multiplications, the number of cycles needed to perform the operation is not fixed but depends on the values of the used operand [23]. Therefore, the execution time varies depending on the current state and input of the filter. In particular, the Kalman filter has a disadvantage here because the number of needed operations as well as the range of the used values is higher compared to the other filters. This results in more operations for the normalization of the data.

### 4.3. Summary for the Extra-Functional Properties

Overall, it can be concluded that the computational effort of the used algorithms can be significantly reduced by using a fixed-point implementation. In particular, the computational effort of the 16-bit fixed-point implementations is around six times lower than their 32-bit floating-point counterpart. Looking at the computational effort alone, fixed-point implementations should be preferred over floating-point implementations. Furthermore, the complementary filter, Madgwick filter, and Mahony filter should be preferred over the Kalman filter.

The reduced computational effort alone, however, does not allow for a definite statement about which fusion algorithm and data format is preferable for a hardware restricted environment. In the following Section 5, this work will examine how much the quality of the used sensor fusion algorithms is influenced by using fixed-point arithmetic and how the quality of the algorithms compares.

## 5. Results: Functional Properties

To assess the usability of the used sensor fusion algorithms and data formats, it is not only important to examine their code size and computational effort. It is obvious that also the quality of the output of the sensor fusion algorithms is essential for its usability. For instance, activity- or gesture detection can be impaired if the quality of the sensor fusion suffers too much from using a different data format. In this section, two investigations about the quality of the sensor fusion will be covered.

In Section 5.1, a general overview about the quality of the used algorithms and data format will be given, including an analysis of the impact of using fixed-point arithmetic. Later, in Section 5.2, an extensive statistical analysis of the impact of algorithm, data format, and movement speed will be presented. Finally, Section 5.3 covers external influences on the result of the sensor fusion algorithms.

### 5.1. General Comparison of the Fusion Results

To get a general overview about the influence of both the used fusion algorithm and the used data format, multiple measurements have been conducted for a direct comparison of the fusion results. Early in the investigation, it was decided to compare the results from the fusion algorithms using only the data from the accelerometer and the gyroscope and not use the data from the magnetometer. Early measurements showed that, in spite of prior calibration of the magnetometer, the data for the *z*-axis showed errors that were too high to allow for a meaningful assessment of the quality of the sensor fusion algorithms.

**Figure 7:**
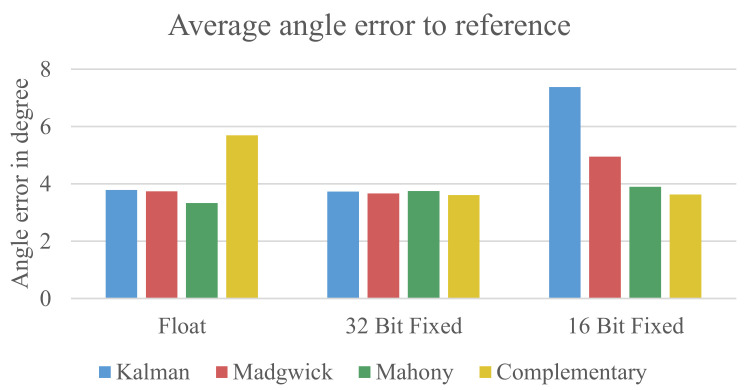
Early error estimation with *z*-axis.

Figure 7 shows the results of the measurements using only the data from the gyroscope and the accelerometer.

It can be seen that the floating-point formats and 32-bit fixed-point formats have around the same error. The complementary filter has a slightly higher error for the floating-point implementation. In the 16-bit fixed-point implementation, the Kalman filter shows a significantly higher error than the other used algorithms. The reason for this error will be described later in Section 5.1.1. Overall, it is evident that the errors for all configurations are relatively high with an average error of 4.14° for the floating-point data, 3.69° for the 32-bit fixed-point data, and 4.90° for the 16-bit fixed-point data.

**Figure 8:**
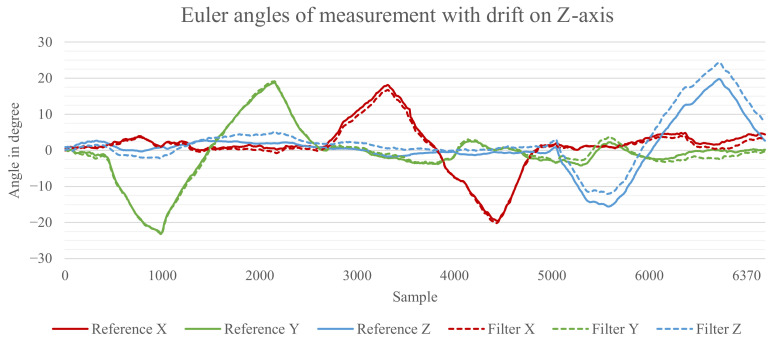
Euler angles of the output from the Madgwick filter with visible drift on the *z*-axis.

The reason for the relatively high errors is that leaving out the data of the magnetometer and therefore the possibility to correct the data from the *z*-axis results in a drift of the *z*-axis, which drastically increases the overall error. Figure 8 displays the rotation for the axes *x*, *y*, and *z* for a measurement where each axis was rotated back and forth to approximately 20° sequentially.

**Figure 9:**
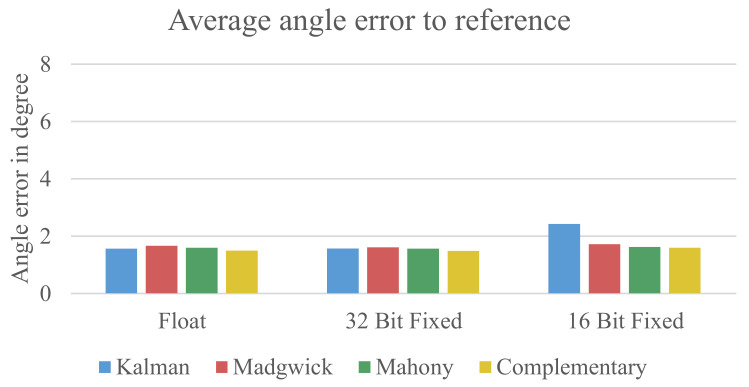
Early error estimation without the *z*-axis.

The solid lines show the rotation measured by the reference system. The dashed lines show the estimation from the Madgwick filter. It can be seen that the *z*-axis of the estimated orientation drifts away from the reference over time. This drift is higher than the average difference found for the *x*- and *y*-axis and superimposes the error we want to measure. Therefore, it was decided to apply a correction for the *z*-axis effectively excluding its errors. The results of the evaluation with a corrected *z*-axis can be seen in Figure 9.

The first obvious difference to the previous evaluation approach is a significantly lower overall error for all configuration replacedbeing wellclearly below 2° except for the 16-bit fixed-point implementation of the Kalman filter. Table 2 shows the average error for all errors from 15 measurements.

The results shown in Table 2 indicate that there is no meaningful difference between the used sensor fusion algorithms and data formats. Interestingly, the results from the 32-bit fixed-point implementation even show a slightly lower error compared to the floating-point implementation. The 16-bit fixed-point variant also does not show significantly higher errors except for the Kalman filter.

#### 5.1.1. Quantization Errors

The results from the general assessment of the differences between the used filters and data formats show two interesting results that will be discussed in this section.

The first finding was that the results from the sensor fusion using 32-bit fixed-point data showed a slightly lower error than their floating-point counterpart. This artifact can be explained by the interaction of two factors. As described in Section 3.2, the data inside a quaternion are always normalized to a length of one, which therefore limits the range of possible values. For the examined filters, the radix point of the data could be set to 7-bits for the integer part and 24-bits for the fractional part. Compared to the standard floating-point implementation, which has 23-bits for the fraction, the 32-bit fixed-point implementation has a 1-bit higher precision.

**Figure 10:**
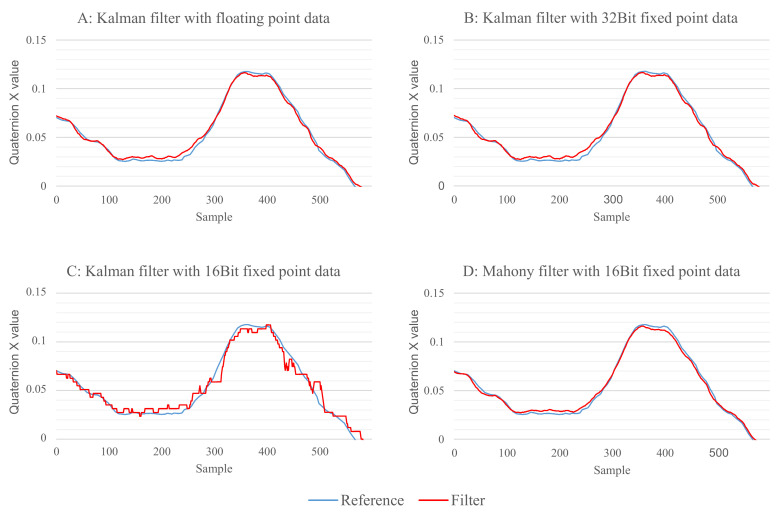
Comparison of the fusion out of the Kalman filter for the three data formats. (**A**) output of the Kalman filter with 32-bit floating-point data; (**B**) output of the Kalman filter with 32-bit fixed-point data; (**C**) output of the Kalman filter with 16-bit fixed-point data; (**D**) output of the Mahony filter with 16-bit fixed-point data.

The second finding is the obviously lower quality of the result from the Kalman filter in the 16-bit fixed-point implementation. Due to the way the Kalman filter works internally, it needs a higher range of values compared to the other three filters. For the Madgwick filter, the Mahony filter, and the complementary filter, it was sufficient to use 3-bits for the integer part and 12-bits for the fraction to be able to cover the range of possible values. The Kalman filter, however, needs the 7-bit integer part that is already used in the 32-bit fixed-point implementation, which only leaves 8-bits for the fractional part. The result of this reduced accuracy can be seen in Figure 10.

Figure 10A,B show the Kalman filter with 32-bit floating-point implementation and 32-bit fixed-point implementation, respectively. There is no visible difference between both results. However, for the 16-bit fixed-point data shown in Figure 10C, one can clearly see quantization errors as a result of the lower precision, which results in a higher overall error. As a comparison, Figure 10D shows the results for the Mahony filter with 16-bit fixed-point data where no quantization errors are visible. The quantization errors are also the reason why the Kalman filter showed a significantly higher error before the correction of the *z*-axis was applied. Without the magnetometer data, the *z*-axis is only changed by the data of the gyroscope and, due to the low precision, all angle rates lower than 90°/s will be rounded to 0. For the performed measurements, the angle rate rarely exceeded this value, resulting in the *z*-axis not showing any rotation change.

### 5.2. Statistical Analysis

The general comparison of the sensor fusion algorithms and data formats already showed that using the 32-bit fixed-point implementation does not have a negative impact on the results of the sensor fusion. Furthermore, using the 16-bit fixed-point implementation also does not decrease the performance of the algorithms in a significant amount for most cases.

To increase the understanding about the impacts of the sensor fusion algorithms, data formats, and also the influence of the human interaction, while conducting the experiments, an extensive statistical analysis has been conducted using the Gage R&R methodology commonly used in the measurement systems analysis [33,34]. As described in Section 3.7, this methodology allows for statistically analyzing the influence of difference factors of the measurement on the overall result. Although normally used to assess the repeatability and reproducibility of a measurement system, the same statistical methods can also be used to determine how algorithm, data format, or average angle rate of the movement can influence the results.

For the analysis, 10 measurements have been conducted for three different angle rates. Ten measurements were made for an average angle rate of 7.5°/s, 10 measurements for 15°/s, and 10 measurements for 30°/s. Using four different fusion algorithms and three data formats, 360 measurement results have been generated. Using different angle rates for the investigation was also an important factor to see if the algorithms behave differently in different scenarios. For instance, the Kalman filter might work better with higher frequencies than the other filters.

The statistical analysis has been divided into two categories to better analyze the impact of different factor on the results.

#### 5.2.1. Results Grouped by Data Format

**Figure 11:**
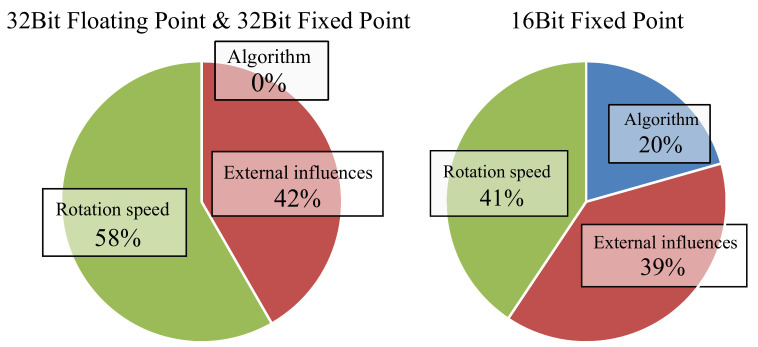
Estimated influence of rotation speed, external influences, and used algorithm on the result of the sensor fusion grouped by data format.

For an analysis on how movement speed and the chosen algorithm influence the outcome of the sensor fusion three statistical analyses have been made for each data formats. The results can be seen in Figure 11.

The results for the 32-bit floating-point implementation and the 32-bit fixed-point implementation show nearly the same result and are therefore grouped into one graph. For the 32-bit floating-point implementation and the 32-bit fixed-point implementation, the statistical analysis showed that around 58% of the differences between the measurements was caused by different rotation speeds and 0% was caused by the chosen algorithm indicating that all algorithms behaved nearly identical for the chosen scenario of human interaction. The factor of external influences which causes 42% of the measurement differences can neither be accounted to the used algorithm, movement speed, or data format. Possible causes for the external influences will be discussed in Section 5.3.

For the 16-bit fixed-point data, the result looks a bit different. It can be seen that the used algorithm influences the overall result by around 20%. The rotation speed has an influence factor of 41% and the external influences have an influence of 39%. This result was expected because it has already been shown in Section 5.1 that the Kalman filter has a reduced accuracy in this configuration.

#### 5.2.2. Results Grouped by Movement Speed

**Figure 12:**
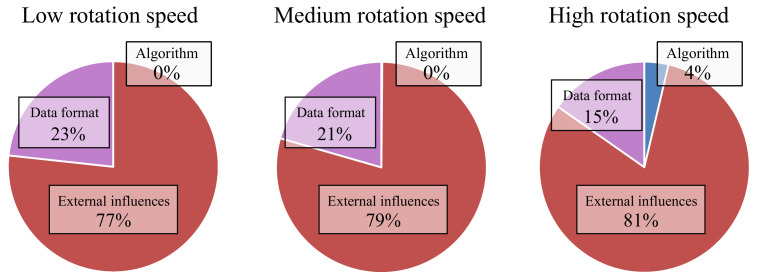
Estimated influence of data format, external influences, and used algorithm on the result of the sensor fusion grouped by rotation speed.

The second part of the statistical analysis aimed to reveal how much the data format influences the overall result compared to the used algorithm. Therefore, the data was grouped by the movement speed. The results can be seen in Figure 12.

For the low and medium movement speeds, the influence of the different factors is roughly the same. The data format influences the results by 23% and 21%. For both, the influence of the used algorithms is at 0%. Most of the variance between the measurements (77% and 79%) cannot be assigned to either the data format or the algorithms.

For a high rotation speed, one can see that the chosen algorithm does have a slight influence on the results. Separating the results by the used data format, it is again evident that the highest error can be observed for the 16-bit fixed-point implementation of the Kalman filter, which was 1.12° higher than the average.

The results show that the filters do not behave differently for the movement speeds that were used for the evaluation. Therefore, all filters appear to be suitable for different scenarios.

### 5.3. Analysis of External Influences

The statistical analysis of the data revealed that a signification amount of the variance between the conducted measurements cannot be accounted to fusion algorithm, data format, or movement speed. Therefore, other factors or external influences contribute to the resulting differences.

#### 5.3.1. Movement Speed

**Figure 13:**
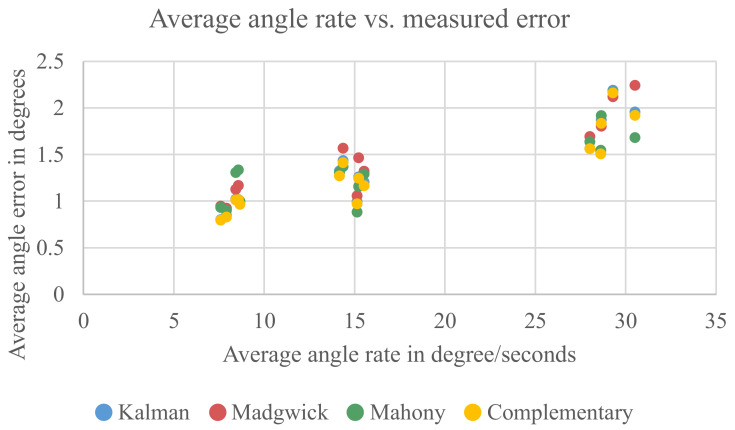
Comparison between the average angle rate of a measurement and the measured error.

The analysis in Section 5.2.1 showed that the rotation speed has a high influence on the results. For the analysis, the measurements have been grouped into three groups with an average angle rate of 7.5°, 15° and 30°. The goal of the work was to evaluate how the fusion algorithms behave when a device is used by humans. Therefore, all measurements have been conducted by a human and will, of course, differ slightly in the conducted movement speed, even for the same group. To see if the different angle rates inside a group contribute to the *external influences*, the average angle rate and calculated errors have been plotted in Figure 13.

One can clearly see that the average error correlates with the average angle rate of the measurement. For the group with the highest angle rate, it is also visible that the movement speed inside this group influences the result. However, for the other two groups, this correlation is not that obvious. It can be concluded that the difference in average angle rate inside a group can only explain a small part of the *external influences*.

#### 5.3.2. User Interaction

**Figure 14:**
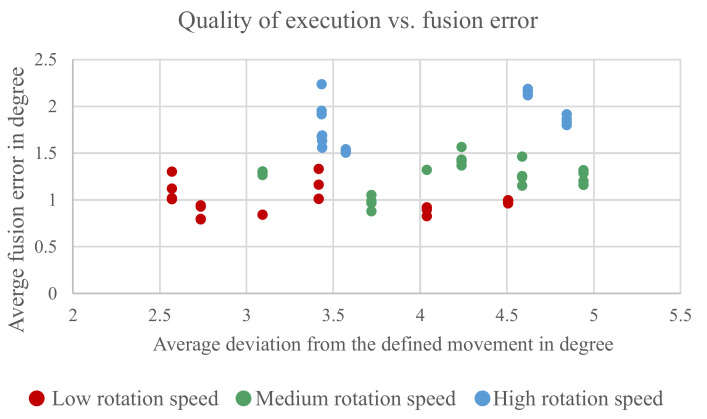
Comparison between accuracy with which the user conducted a predefined movement and error from the sensor fusion output.

Another possible factor is the interaction with the human who conducted the measurements. The used measurement method from [31] uses a graphical user interface to instruct the probationer on what movements have to be done and displays in real time how well the probationer follows these movements. This should ensure that the measurements all depict the same movements to be comparable. However, as already seen in Figure 13, it is not always possible to follow exactly the predetermined movements. To determine if the variation in the conducted movements contributes to the *external influences*, it has been analyzed if there is a correlation between the accuracy with which the probationer followed the predetermined movements and the measured errors. The results can be seen in Figure 14.

The figure shows the measured error on the *x*-axis and the average difference between the predetermined movement and the movement probationer actually did on the *y*-axis. There is no clear correlation between precision of the conducted movement to the measured error. One can see a tendency that the measurements with the higher angle rates are also the ones with a higher deviation from the predetermined movement; however, this is more a correlation with the higher angle rates because it becomes more difficult to follow the faster movements.

#### 5.3.3. Other Factors

The previous sections showed that the different angle rates can be made accountable for a small amount of the *external influences* seen in the statistical analysis.

Other factors that can contribute to the *external influences* are the measurement system itself and the used inertial sensors.

For the measurement system, the following factors can contribute:

- Precision of the image analysis. This factor is influenced by the resolution of the camera, the frame rate of the camera, and the precision of the used image analysis algorithm.
- Cross correlation of the data from reference and sensor fusion. When the timestamps of the data do not fit precisely, there will be an error added to the whole measurement.

For the inertial sensors, the error properties of the sensors like bias, temperature drift, scaling error, and noise can influence sensor fusion. These properties can also change slightly between measurements.

**Figure 15:**
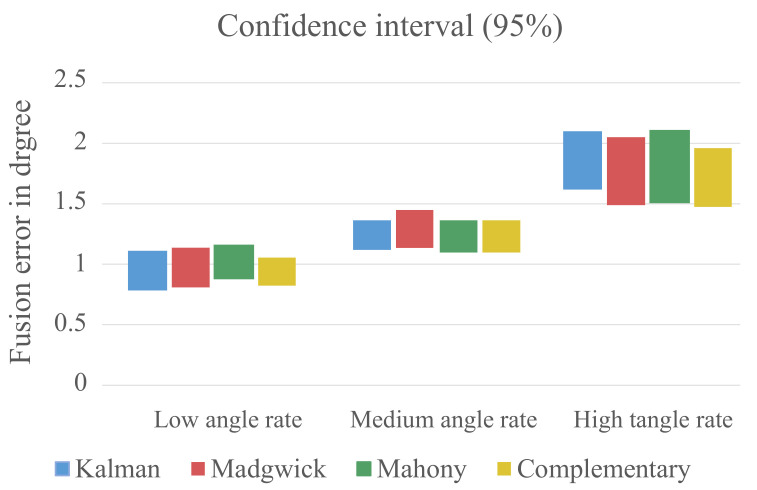
Confidence interval of the captured data grouped by the three angle rate groups.

Since the mentioned factors cannot be ruled out, the confidence interval of the measured data from each algorithm was calculated to see if the variance of the measurements negatively influences the possibility to make a statement about the fitness of the assessed algorithms. The results can be seen in Figure 15.

The confidence intervals for the algorithms are relatively small, and there is only a small overlap between the different angle rate groups. This is a strong indicator that the variation of the measured data is low enough to make a statement about the fitness of the assessed algorithms and to distinguish between the three groups with different movement speeds.

## 6. Discussion

In the work at hand, it was shown that using fixed-point arithmetic can reduce the computational effort of the examined algorithms by around 50% for 32-bit fixed-point data and around 80% for 16-bit fixed-point data compared to a standard implementation with 32-bit floating-point data.

Using a 32-bit fixed-point implementation does not negatively impact the result of the sensor fusion algorithms. Even the 16-bit fixed-point implementations delivered usable results and can be used too, although with a slight decrease in precision.

An exception is the Kalman filter, which had inferior results using the 16-bit fixed-point implementation and consistently requires around three times the computation time needed by the other algorithms. Hence, this filter is not recommended for a hardware restricted environment, considering that our work and the authors of [12,13,14] concluded that Madwick filter and Mahony filter show a similar quality.

The reduction of the computational effort significantly influences possible use cases and power consumption. The 32-bit floating-point version of the Kalman filter cannot handle data faster than 250 Hz and would cause a high power consumption close to the maximum of 5.9 mA of the used μC. Using, for example, the 32-bit fixed-point version of the Madwick filter would allow for data rates above 1700 Hz or only require approximately 14% of the power consumed by the Kalman filter. For a hardware restricted environment with limited power availability, a 32-bit fixed-point implementation and simpler fusion algorithm are therefore recommended.

In the statistical analysis of the sensor fusion results, we were able to show that most combinations of data formats and algorithms were usable. However, the analysis revealed a high influence from other factors aside from the used algorithm, rotation speed, and data format. The cause and impact of these factors are a possible target for further studies.

## Figures and Tables

**Table 1:** Average execution time per sensor sample needed by the sensor fusion algorithms.

Data Format	Kalman	Madgwick	Mahony	Complementary
32-bit Floating-Point	3.963 ms	1.142 ms	0.758 ms	0.782 ms
32-bit Fixed-Point	1.923 ms	0.560 ms	0.350 ms	0.382 ms
16-bit Fixed-Point	0.621 ms	0.166 ms	0.121 ms	0.123 ms

**Table 2:** Average error in degree measured for the four sensor fusion algorithms using the three data formats.

Data Format	Kalman	Madgwick	Mahony	Complementary
32-bit Floating-Point	1.557	1.657	1.588	1.489
32-bit Fixed-Point	1.562	1.603	1.557	1.478
16-bit Fixed-Point	2.429	1.722	1.626	1.539

## Abbreviations

The following abbreviations are used in this manuscript:

AHRS	Attitude and Heading Reference System
BLE	Bluetooth Low Energy
MEMS	Micro-Electro-Mechanical Systems
μC	Microcontroller
IMU	Inertial Measurement Unit
SiP	System in Package
ROM	Read Only Memory
SRAM	Static Random-Access Memory
